# The prevalence and heterogeneity of prehypertension: a meta-analysis and meta-regression of published literature worldwide

**DOI:** 10.5830/CVJA-2011-058

**Published:** 2012-02

**Authors:** X Guo, X Zhang, J Hu, Y Sun, L Zheng, L Zou, J Li, Z Sun

**Affiliations:** Department of Cardiology, First Affiliated Hospital of China; Medical University, Shenyang, People’s Republic of China; Department of Cardiology, First Affiliated Hospital of China; Medical University, Shenyang, People’s Republic of China; Department of Cardiology, First Affiliated Hospital of China; Medical University, Shenyang, People’s Republic of China; Department of Cardiology, First Affiliated Hospital of China; Medical University, Shenyang, People’s Republic of China; Department of Clinical Epidemiology, Library, Shengjing; Hospital of China Medical University, Shenyang, People’s Republic of China; Department of Preventive Medicine, Tongji University, Shanghai, People’s Republic of China; Heart, Lung and Blood Vessel Centre, Tongji University, Shanghai, People’s Republic of China; Department of Cardiology, Shengjing Hospital of China; Medical University, Shenyang, People’s Republic of China

**Keywords:** epidemiology, meta-analysis, meta-regression, prehypertension, prevalence

## Abstract

**Objective:**

Prehypertension appears to be a precursor of hypertension and has been recognised as a major risk factor for cardiovascular disease (CVD). Recognition of prehypertension provides important opportunities for preventing hypertension and CVD. We aimed to investigate the world-wide prevalence and heterogeneity of prehypertension.

**Methods:**

We performed a meta-analysis of cross-sectional studies worldwide that reported the prevalence of prehypertension. We searched for publications between January 1966 and November 2010, using PubMed, Ovid and the Cochrane Library, with the keyword ‘prehypertension’, supplemented by a manual search of references from recent reviews and relevant published original studies. Pooled prevalence of prehypertension was calculated using random-effects models. Heterogeneity was investigated by subgroup analysis and meta-regression. Twenty-two articles met our inclusion criteria, with a total sample of 242 322 individuals.

**Results:**

The overall pooled prevalence of prehypertension was 38%. Significant heterogeneity across estimates of prevalence was observed (*p* = 0.000, *I*^2^ = 99.9%). The prevalence rose as the sample size increased, and was higher among men than women (41 vs 34%). The non-Asian population was more likely to be prehypertensive than Asian individuals (42 vs 36%). A high prevalence of 47% was observed among the black African population in the non-Asian subgroup. The inception year of the surveys was the only source of heterogeneity we found by meta-regressional analysis (*p* = 0.06).

**Conclusion:**

These results indicate that the prevalence of prehypertension was relatively high, particularly among males. Although more attention has been paid to this segment of the population since 2003, additional practical and reasonable steps should be taken to prevent and treat prehypertension.

## Abstract

High blood pressure (BP) has always been recognised as a major risk factor for cardiovascular disease (CVD).^1^,^2^ In 2003, the Joint National Committee on Prevention, Detection, Evaluation, and Treatment of High Blood Pressure first introduced the blood pressure category ‘prehypertension’, replacing former categories of ‘high normal’ and ‘above-optimal’ blood pressure.^3^ Individuals with systolic blood pressure (SBP) between 120 and 139 mmHg or diastolic blood pressure (DBP) between 80 and 89 mmHg were categorised as having prehypertension. This evidence-based definition was established to focus clinical and public health attention on the population who are at higher-than-normal CVD risk, since prehypertension has been noticed to be associated with carotid artery stenosis, myocardial infarction, coronary artery disease and many other adverse consequences.^4^-^6^

Effective strategies on prevention for this segment of the population would be of value. Because screening and treatment would be based on the new classification, it is important to know the prevalence of this new blood pressure category. However, the prevalence varies considerably worldwide. Therefore, we performed this study, aiming to systematically review the findings of all available studies and estimate the overall prevalence of prehypertension. In addition, we aimed to explore potential sources of heterogeneity.

## Methods

We performed a systematic search for publications between January 1966 and November 2010 in which the prevalence of prehypertension was reported, using PubMed, Ovid and the Cochrane Library. Studies were identified using the keyword ‘prehypertension’. Further information was retrieved by a manual search of references from recent reviews and relevant published original studies. The reference lists of the articles obtained were screened in order to identify other relevant references, which were then retrieved.

We included cross-sectional studies that reported the prevalence of prehypertension among individuals over 15 years of age. It had to be original research written in English and Chinese, containing the minimum information necessary to conduct pooled analysis of prevalence. There had to be a clear definition of prehypertension according to the seventh annual report of the Joint National Committee (JNC7): SBP between 120 and 139 mmHg or DBP between 80 and 89 mmHg.

Studies were excluded if the participants were limited to a particular occupation (e.g. teachers) and population (e.g. children or adolescence), or if there were multiple reports of the same results. In addition, we excluded studies using non-JNC7 standards (e.g. normal/high-normal BP) to avoid inconsistency of outcomes resulting from different blood pressure categories.

To avoid bias in the data abstraction process, two authors independently collected the data from the articles and compared results, with disagreements resolved by discussion among the investigators. We extracted from the full-text articles the study and population characteristics such as publication year, country of data collection, characteristics of target population (e.g. baseline age, gender and sample size), methods of blood pressure measurement (e.g. mercury sphygmomanometer), prevalence of prehypertension and quantitative data (i.e. raw numbers and percentages) related to the meta-analysis.

## Statistical analysis

We calculated crude prevalence and standard errors. Pooled prevalence was estimated by Stata statistical software package (version 11.0) using the command ‘metan’ with the use of a random-effects model, which allows for heterogeneity of effects between studies.^7^ The heterogeneity among studies was tested by Cochran’s *Q* test.^8^ The quantity *I*^2^ that describes the percentage of variation across studies included was calculated.^9^,^10^

We conducted subgroup analyses to examine potential sources of heterogeneity according to: gender, region, sample size, year of inception of the survey, and method of blood pressure measurement. The mean age was excluded because this variable was only available for half of the studies. We also tested the heterogeneity by conducting a meta-regression analysis.

To study possible publication bias, we evaluated funnel plots. A deficiency in the base of the funnel with asymmetry indicates the presence of possible publication bias from unpublished small studies. Publication bias was also assessed by two formal tests: Begg’s adjusted-rank correlation test and Egger’s regression asymmetry test. For all tests, a probability level less than 0.1 was considered significant. All statistical analyses were performed with Stata software 11.0.

## Results

**Fig. 1:**
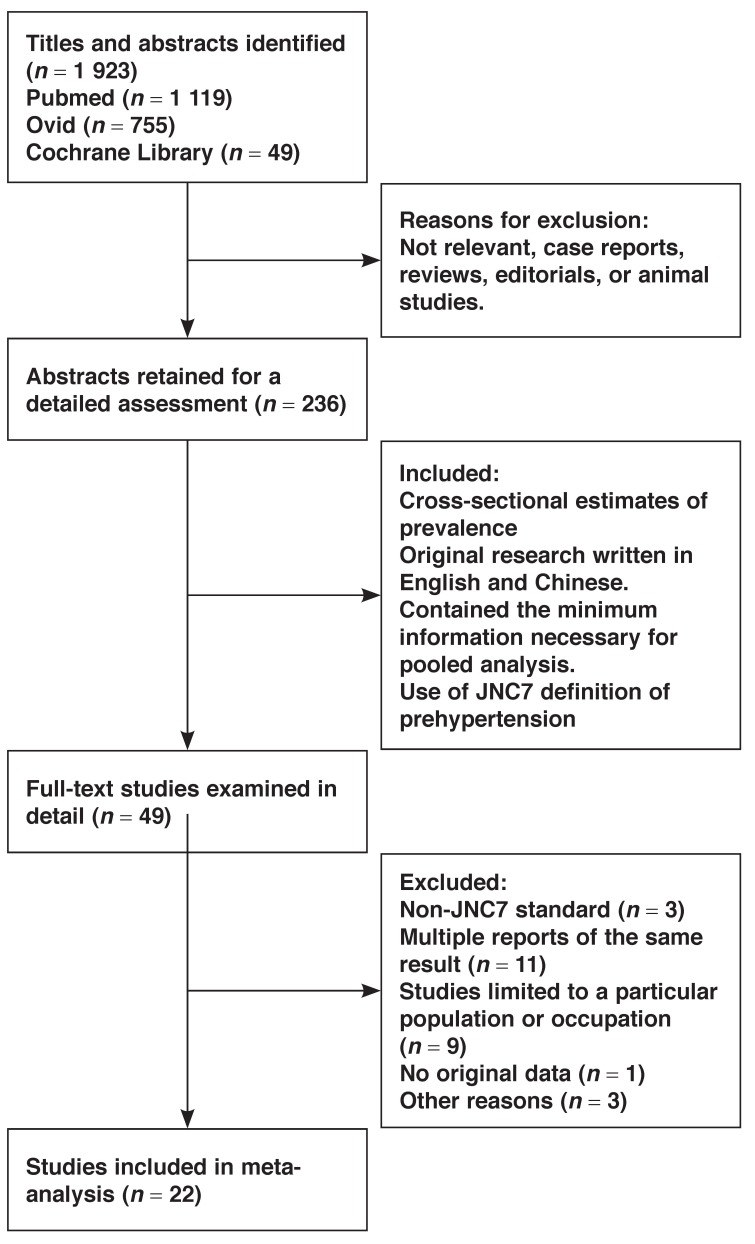
Flow chart showing the process of selection of the relevant studies.

The literature searches yielded 1 923 articles. We assessed abstracts and titles against the inclusion criteria, and selected 49 articles for a detailed examination. After reviewing the full texts, a total of 22 studies^11^-^32^ met our inclusion criteria. Fig. 1 shows the selection process and reasons for excluding studies.

Our analysis included a total sample of 242 322 individuals (126 899 females and 115 423 males). The studies included were all published recently, the majority of which after 2005. The sample sizes varied from 782 to 69 722 participants. Most studies were distributed in Asia, including China, Japan, India, Iran, Turkey and Korea. There were also studies from non-Asian countries, such as the USA, Netherlands, Jamaica, Nigeria and Ghana. Characteristics of studies included in the analyses are described in Table 1.

**Table 1:** Characteristics Of 22 Cross-Sectional Studies, Reporting Prevalence Of Prehypertension

*First author, publication year*	*Country*	*Sample size*	*Gender (male %)*	*Age (year)*	*Prevalence of prehypertension (%)*	*Methods of BP measurement*	*Criteria for prehypertension*
Aekplakorn 2008^11^	China	39290	48.2	≥ 15	32.8	mercury sphygmomanometer	JNC7
Agyemang 2007^12^	Netherlands	1432	41.1	35–60	32.8	automated digital BP device	JNC7
Agyemang 2008^13^	Ghana	1431	45	≥ 18	40.0	automated digital BP device	JNC7
Chockalingam 2005^14^	India	2007	75	18–86	47.4	mercury sphygmomanometer	JNC7
Choi 2006^15^	Korea	6074	43.1	≥ 20	31.6	mercury sphygmomanometer	JNC7
Erem 2009^16^	Turkey	4809	45.9	≥ 20	14.5	aneroid sphygmomanometer	JNC7
Ferguson 2008^17^	Jamaica	1972	33.5	15–74	30.0	NA	JNC7
Glasser 2010^18^	US	9799	49.8	≥ 45	56.7	aneroid sphygmomanometer	JNC7
Gupta 2010^19^	US	10380	52.3	≥ 20	36.3	mercury sphygmomanometer	JNC7
Isezuo 2010^20^	Nigeria	782	52.3	15–65	58.7	automated sphygmomanometer	JNC7
Ishikawa 2008^21^	Japan	12048	39.1	18–90	33.0	automated digital BP device	JNC7
Janghorbani 2008^22^	Iran	69722	50.3	25–65	52.1	mercury sphygmomanometer	JNC7
Kawamoto 2008^23^	Japan	2841	42.5	19–90	25.3	automated digital BP device	JNC7
Li 2008^24^	China	2589	41.1	20–84	38.4	mercury sphygmomanometer	JNC7
Lin 2009^25^	China	6204	42.7	61.7 ± 11.9	30.2	mercury sphygmomanometer	JNC7
Ling 2008^26^	China	5272	48.2	≥ 15	36.3	mercury sphygmomanometer	JNC7
Sit 2010^27^	China	1448	54.4	35–74	42.7	mercury sphygmomanometer	JNC7
Sun 2007^28^	China	29970	50.5	35–99	47.0	electric sphygmomanometer	JNC7
Tsai 2005^29^	China	2225	46.7	18–96	34.0	standardized sphygmomanometer	JNC7
Yadav 2008^30^	India	1112	50.1	49.8 ± 11.5	32.3	mercury sphygmomanometer	JNC7
Yang 2010^31^	China	20167	38.5	35–74	54.6	mercury sphygmomanometer	JNC7
Yu 2008^32^	China	10748	47.0	35–74	21.9	mercury sphygmomanometer	JNC7

NA = not available; BP = blood pressure.

## Pooled results from the meta-analysis

**Fig. 2:**
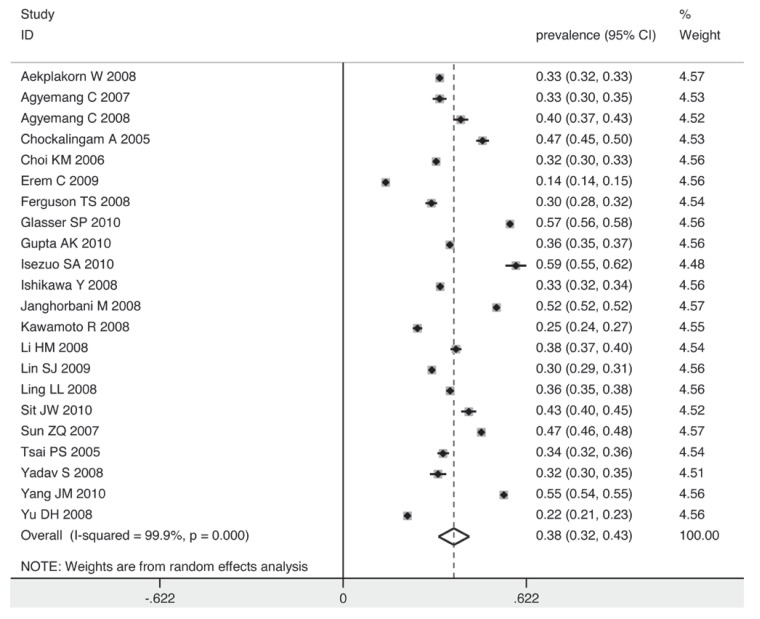
Pooled prevalence of prehypertension according to 22 cross-sectional studies.

The prevalence of prehypertension varied widely, from 14.5% in the study by Erem *et al.*^16^ to 58.7% in the study by Isezuo *et al.*^20^ Fig. 2 shows the overall estimates of prevalence and 95% confidence intervals (CI) from the individual countries. Meta-analysis of all 22 studies yielded an overall pooled prevalence of 38% (95% CI: 32–43%), with substantial heterogeneity observed (χ^2^ = 14430.35, *p* = 0.000, *I*^2^ = 99.9%).

## Sources of heterogeneity

**Fig. 3:**
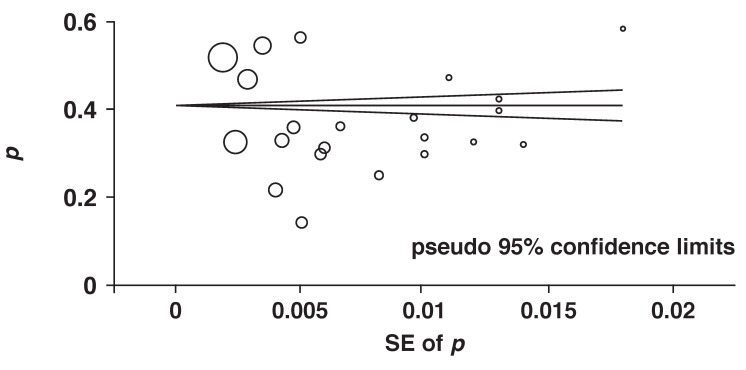
Begg’s funnel plot of 22 cross-sectional studies.

Fig. 3 shows a Begg’s funnel plot for the visual assessment of publication bias. A symmetrical pattern was observed, indicating the absence of publication bias. In addition, both Begg’s adjusted-rank correlation test and Egger’s regression asymmetry test showed no evidence of substantial publication bias (*p* = 0.259 for Begg’s test; *p* = 0.159 for Egger’s test).

Meta-regression analyses showed that gender ratio (*p* = 0.112), sample size of the survey (*p* = 0.179), region of the study (*p* = 0.242) and method of blood pressure measurement (*p* = 0.942) were not associated with heterogeneity (Table 2). The only source of heterogeneity across the studies, identified with meta-regression analyses, was the year of inception of the survey (*p* = 0.06).

**Table 2:** Results Of The Meta-Regression Model

	*Coefficient*	*Standard error*	p-*value*
Gender (male ratio)	0.0049	0.0029	0.112
Start of survey	0.0106	0.0053	0.06
Sample size	2.06E–06	1.48E–06	0.179
Region	–0.065	0.054	0.242
Method of BP measurement	0.0041	0.0553	0.942

**Fig. 4:**
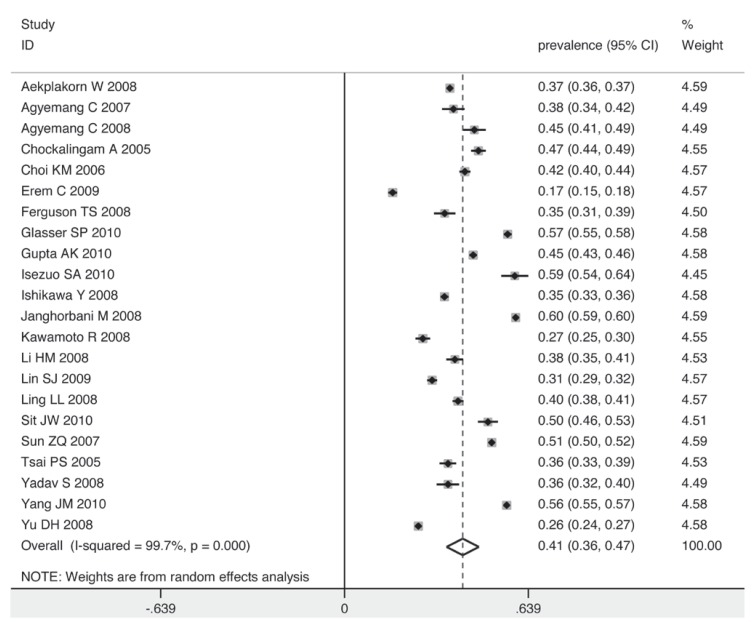
Pooled prevalence of prehypertension in males according to 22 cross-sectional studies.

**Fig. 5:**
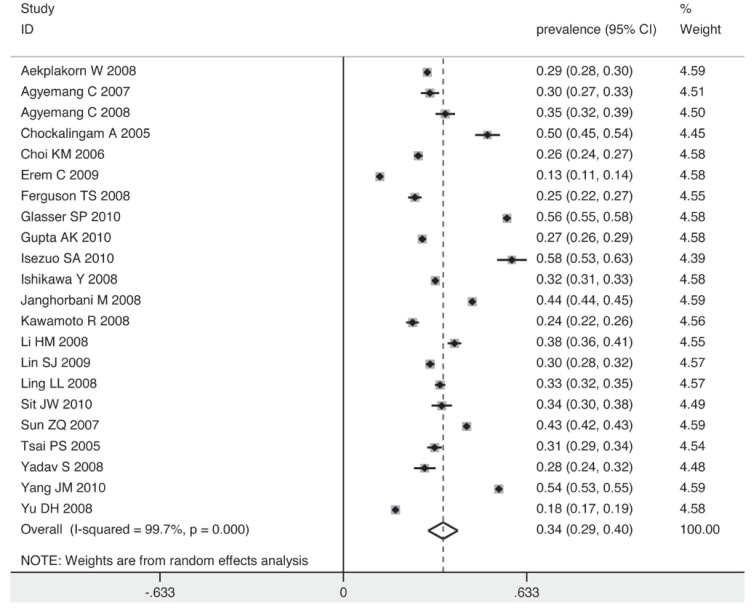
Pooled prevalence of prehypertension in females according to 22 cross-sectional studies.

**Fig. 6:**
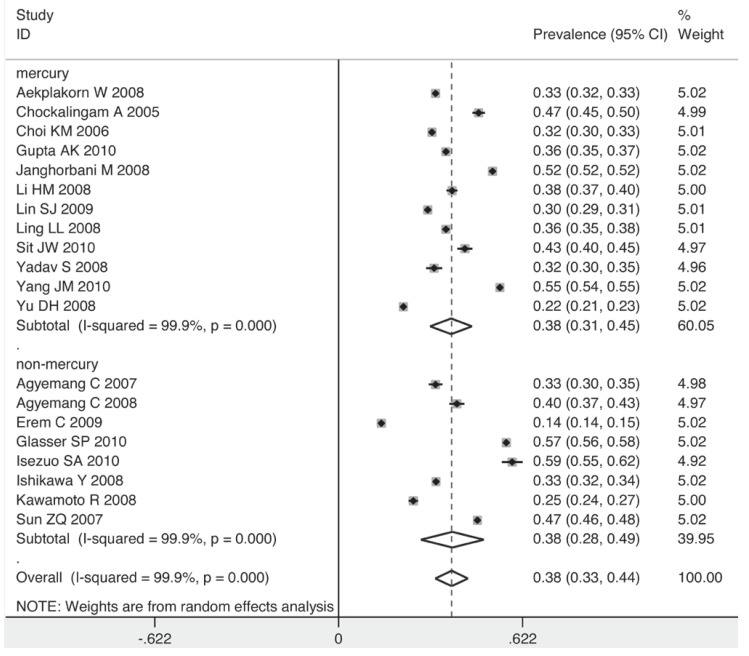
Pooled prevalence grouped by method of BP measurement.

To explore the sources of heterogeneity graphically and confirm the results of the meta-regression, five subgroups were analysed. The prevalence of prehypertension was higher among men than women (41 vs 34%) (Fig. 4) and (Fig. 5). Corresponding with the result of the meta-regression, the method of blood pressure measurement had no influence on the prevalence of prehypertension (Fig. 6). The prevalence rose as the sample size increased.

**Fig. 7:**
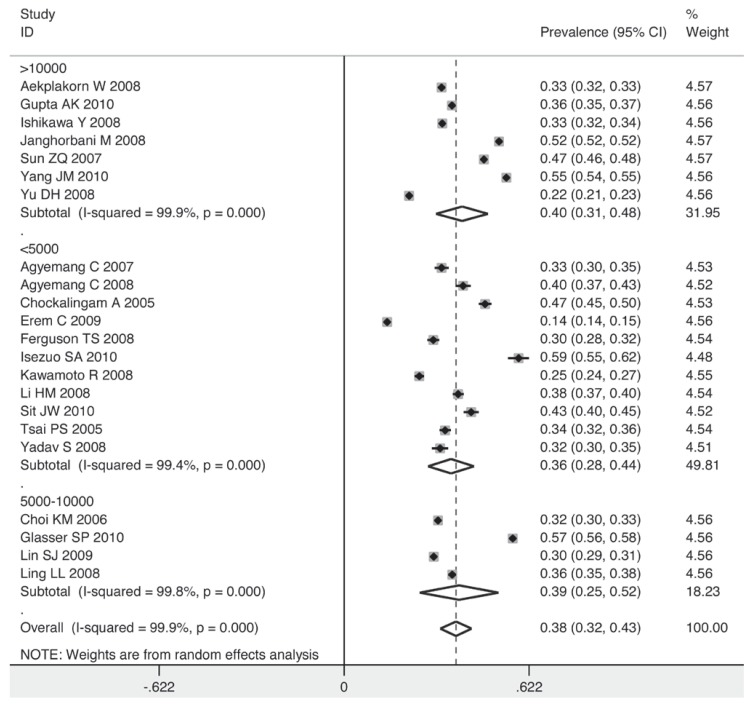
Pooled prevalence grouped by sample size.

**Fig. 8:**
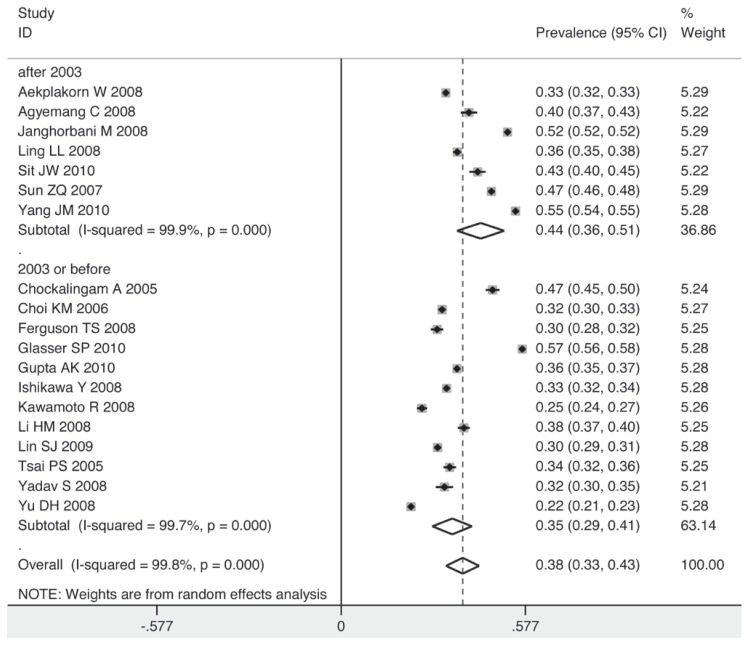
Pooled prevalence grouped by inception year of survey.

**Fig. 9:**
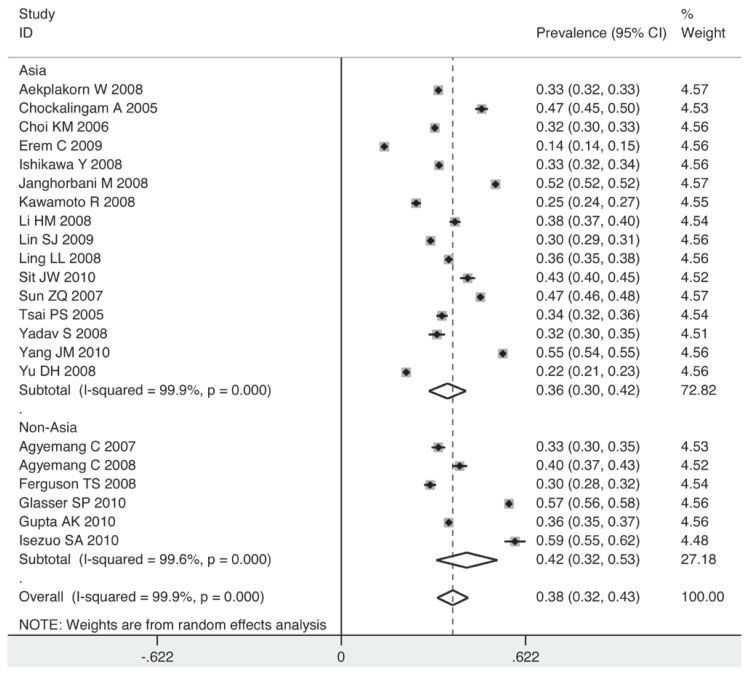
Pooled prevalence grouped by Asian and non-Asian population.

**Fig. 10:**
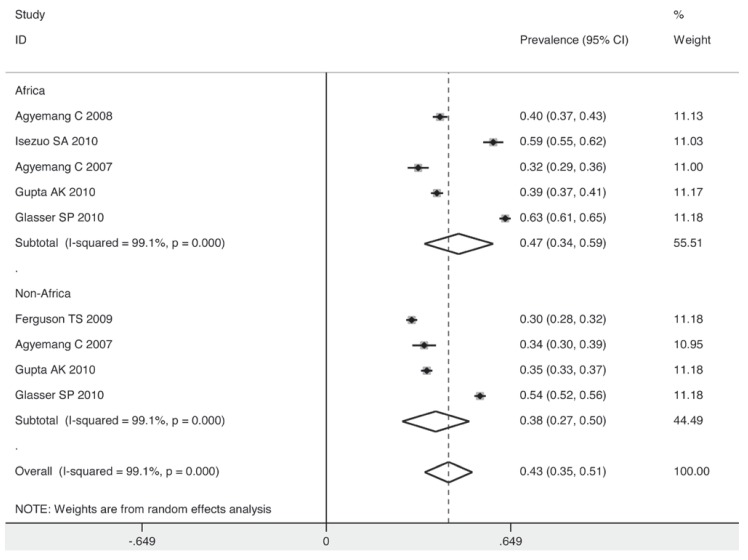
Pooled prevalence grouped by African and non-African population.

The prevalence for the subgroup with a sample size over 10 000 individuals was 40%, while for that with a sample size below 5 000, it decreased to 36% (Fig. 7). The studies taking place in 2003 or before had a lower pooled estimate than those after 2003 (35 vs 44%) (Fig. 8). Individuals in non-Asian countries were more likely to be prehypertensive than their counterparts in Asia (42 vs 36%) (Fig. 9). In the non-Asian subgroup, the prevalence of prehypertension among the black African population was 9% higher than in the non-African population (mainly white) (Fig. 10).

## Discussion

In the 22 cross-sectional studies included, the prevalence of prehypertension varied widely. Considering that prehypertension has developed a more important status in recent years, we presumed that an overall estimate of prevalence by meta-analysis would be necessary for further strategies of prevention or treatment of prehypertension.

Our result showed that the overall pooled prevalence of prehypertension among individuals aged 15 years and older was 38% (95% CI; 32–43%). As a precursor of hypertension, this prevalence is relatively high, and effective approaches for prevention and treatment are needed in order to reduce the development of hypertension and CVD in this segment of the population.

Gender-related and age-dependent differences in blood pressure levels have been the subject of multiple clinical and epidemiological studies over the past decades.^33^-^36^ The risk of hypertension in females increases with age and becomes more prevalent among women after 59 years of age than among men, probably due to changes related to the menopause.^37^ The mechanisms involved are complicated, including activation of the renin–angiotensin system, the impact of progesterone and aldosterone, leptin levels and the regulation of oestrogen.^38^-^42^

The prevalence of prehypertension would be expected to be higher among women after menopause, and the difference in prevalence between older men and women may be less obvious. We found in our analysis however that the prevalence of prehypertension was higher among males than females (41 vs 34%). Although a pooled analysis of different age groups was not conducted due to insufficient data, we concluded that males are generally more at risk for prehypertension than females.

Within our expectation, a large variation in estimates of prevalence of prehypertension was found. This variability was not explained by gender ratio, sample size of the survey, region of the study or method of blood pressure measurement. However, some of these variables did change the estimated prevalence of prehypertension.

The maximum and minimum prevalence rates of prehypertension were observed in Nigeria and Turkey, respectively. The pooled prevalence was higher among non-Asian individuals (42%) than in the Asian population (36%). In the non-Asian subgroup, a high prevalence of 47% was observed among the African population, possibly due to ethnic diversity and differences in lifestyle.

The sample size of the survey was important when determining prevalence of prehypertension. When the sample size was less than 5 000 individuals, the pooled prevalence was observed to be 36%. As the sample size increased, the prevalence rose accordingly (39% for group of 5 000–10 000 individuals and 40% for group of > 10 000). This positive correlation implies an even higher prevalence of prehypertension among the world population.

The year of inception of the survey was the only source of heterogeneity we found in this pooled analysis. A sharp increase in the prevalence of prehypertension was found after 2003 in the subgroup analysis. Since the JNC7 introduced the term ‘prehypertension’ in their 2003 report, this may have been one of the factors increasing awareness of the risks related to prehypertension at the population level, particularly via the media and in the public health community. In addition, changes in lifestyle in recent years, which contribute to increased body mass, may also result in the increased prevalence of prehypertension.^43^-^45^

The reclassification of blood pressure levels in the new guidelines of the JNC7 in 2003 was prompted by several influential epidemiological studies and clinical trials. It was necessary to know the overall prevalence of prehypertension for the establishment of the new guidelines and to indicate the importance of prevention and treatment strategies among healthcare providers. Morbidity and mortality as well as healthcare costs attributable to prehypertension are substantial.

The rate of progression from prehypertension to clinical hypertension, which depended on blood pressure level and age, was 19% over four years.^46^ Prehypertension was related to a 27% increase in all-cause mortality and a 66% increase in CVD mortality.^47^ In the NHANES-I cohort with a follow up of over 20 years, an estimated 3.4% of hospitalisations and 9.1% of deaths could be attributed to prehypertension.^48^

Unlike hypertensive patients, there is no evidence that antihypertensive medications are beneficial for patients with prehypertension. However, it is evident that lifestyle modification (e.g. weight loss, reduced sodium intake and regular physical activity) can be very successful in reducing blood pressure levels. Therefore, the JNC7 report states that all patients with prehypertension should be ‘firmly and unambiguously advised to practice lifestyle modification’.^3^

Several limitations of the study require consideration. First, analyses were based on cross-sectional studies, in which the inherent limitations may have affected our findings. Second, this meta-analysis was performed on a limited number of articles (*n* = 22), which may have been too small to generate sufficient statistical power for conducting meta-regression analyses. The possibility of residual confounding or bias cannot be excluded. In the meta-regression models, age was not included due to insufficient data, and characteristics of residents (e.g. rural or urban) could not be assessed from the majority of publications. These factors may have played an important role in the variability of the estimates of prevalence. In addition, considering the disagreement on quality criteria for assessing cross-sectional studies, we did not include assessment of the methodological quality of the studies.

## Conclusion

We conducted a meta-analysis of prehypertension prevalence studies, and a meta-regression to investigate the sources of variability across estimates of prevalence. In our study, we found that the overall pooled prevalence of prehypertension worldwide was 38%. This result may be an underestimate since a positive correlation between sample size and prevalence was observed. The prevalence among males was higher than that in females (41 vs 34%), and non-Asians, particularly the black African population, were more likely to be prehypertensive than Asian individuals. The year of inception of the survey was the only source of heterogeneity we found on meta-regression analysis. Further research with sufficient data is expected.
